# Detection of KRAS mutant alleles in circulating cell-free tumor DNA in colorectal cancer patients resistant to cetuximab

**DOI:** 10.1186/1878-5085-5-S1-A42

**Published:** 2014-02-11

**Authors:** Marzia Del Re, Paola Ulivi, Alessandro Passardi, Wainer Zoli, Romano Danesi, Dino Amadori

**Affiliations:** 1 Dept. of Clinical and Experimental Medicine, University of Pisa, Italy; 2 Bioscience and Medical Oncology Units, Istituto Scientifico Romagnolo per lo Studio e la Cura dei Tumori (IRST), IRCCS, Meldola, Italy

## Background

Cancer cells undergoing necrosis or apoptosis release fragmented, low-molecular weight DNA into the circulation, which may be recovered from plasma and processed for molecular analysis. This minimally invasive approach represents a useful alternative to tumor biopsy and a potential application in therapeutic interventions is the periodic monitoring of circulating cell-free tumor DNA for the identification of molecular changes associated with resistance to target-specific treatments or when the mutational status of tumor tissue is not available.

## Scientific objectives

The present study screened KRAS mutant alleles in cell-free circulating tumor DNA in a small cohort of patients resistant to cetuximab treatment to investigate the potential occurrence of KRAS-mutated cell clones arising in tumors as a consequence of selective pressure exerted by cetuximab on KRAS wild-type cancer.

## Technological approaches

Three patients were judged to have a KRAS wild-type primary tumor by standard molecular analysis. These patients received standard chemotherapy with cetuximab and eventually progressed on treatment. At the time of radiologic evidence of disease progression they were screened for the developent of KRAS somatic mutations as a mechanism of secondary resistance. Peripheral blood samples (6 ml) were drawn at pre-cetuximab time-point (baseline) and at PD during cetuximab administration. DNA was extracted from plasma with QIAamp Circulating Nucleic Acid Kit to recover DNA fragments of ≤1000 bp. PCR amplification was carried out with a QX100™ ddPCR™ System (Bio-Rad) on 20 μL-samples containing cftDNA and TaqMan probes for KRAS G12D (35G>A) and G12V (35G>T) labeled with FAM/VIC. Samples were then loaded into a droplet reader, which discriminates the difference in fluorescence amplitudes on the basis of target gene amplification.

## Results interpretation

Analysis of the cell-free tumor DNA samples at PD in patient 1 and 2 showed the presence of the KRAS mutation G12V while in patient 3 the G12D mutation was detected. Interestingly, pre-cetuximab cell-free tumor DNA was positive for KRAS mutant alleles for patients 2 and 3, but was not sufficient to test the mutational status of KRAS in patient 1.

## Outlook and expert recommendations

ddPCR is a third-generation PCR technique for highly sensitive detection of DNA mutations, which has important application in the monitoring of patients for the occurrence of secondary mutations that render their tumors resistant to target-specific anticancer agents.

